# Successful management of cardiac tamponade due to right ventricular perforation following temporary pacing wire removal after primary percutaneous coronary intervention at a district general hospital: a case report

**DOI:** 10.1093/ehjcr/ytag590

**Published:** 2026-08-08

**Authors:** Maria Gabriel, Ioannis Vasiliadis

**Affiliations:** Department of Cardiology, Hereford County Hospital, Wye Valley NHS Trust, Stonebow Road, Hereford HR1 2ER, UK; Department of Cardiology, Hereford County Hospital, Wye Valley NHS Trust, Stonebow Road, Hereford HR1 2ER, UK

**Keywords:** Temporary pacing, Right ventricular perforation, Cardiac tamponade, District general hospital, Primary percutaneous coronary intervention, Agitated saline contrast echocardiography, Case report

## Abstract

**Background:**

Temporary transvenous pacing is frequently required in inferior ST-elevation myocardial infarction (STEMI) complicated by high-grade atrioventricular block. Right ventricular perforation is a rare but life-threatening complication that may present after wire removal rather than at insertion, complicating recognition. Management is particularly challenging in district general hospitals (DGHs) without on-site cardiothoracic surgery.

**Case summary:**

A 62-year-old woman presented in cardiogenic shock to a DGH with inferior STEMI and complete atrioventricular block requiring emergency temporary transvenous pacing and primary percutaneous coronary intervention (PPCI) to the right coronary artery. Approximately 15 min after pacing wire removal, she developed cardiac tamponade with rapid re-accumulation of pericardial effusion despite pericardiocentesis. Repeat coronary angiography excluded coronary perforation. Bedside agitated saline contrast echocardiography in subcostal views confirmed right ventricular perforation by demonstrating echogenic microbubbles within the pericardial effusion. She was stabilized with auto-transfusion and inotropic support and transferred for emergency surgical repair at a regional tertiary cardiothoracic centre. She achieved complete cardiac recovery with preserved biventricular function at long-term follow-up.

**Discussion:**

This case illustrates that life-threatening procedural complications can be successfully managed in DGHs delivering PPCI without co-located cardiothoracic services. The decisive factors were prompt recognition, systematic diagnostic re-evaluation, application of an established bedside diagnostic technique, aggressive haemodynamic stabilization including auto-transfusion, and rapid escalation via direct consultant-to-consultant communication. This integrated approach was critical in achieving an excellent patient outcome. Standing transfer agreements with regional cardiothoracic centres would further reduce time to definitive surgery in comparable emergencies.

Learning points

- Right ventricular perforation following temporary transvenous pacing may cause cardiac tamponade after wire removal rather than at insertion.
- Bedside agitated saline echocardiography is a rapid, point-of-care means of confirming right ventricular perforation when computed tomography (CT) imaging is not feasible.
- Mechanical complications of primary percutaneous coronary intervention can be successfully managed in district general hospitals through prompt diagnosis, auto-transfusion, and close tertiary links.

## Introduction

Inferior ST-elevation myocardial infarction (STEMI) is frequently complicated by high-grade atrioventricular block, sometimes requiring temporary transvenous pacing.^1^ Right ventricular (RV) perforation may progress to cardiac tamponade, with reported tamponade incidence below 1%,^2^ and may present after wire removal rather than at insertion, complicating recognition.^3^ These challenges are amplified in district general hospitals (DGHs) without on-site cardiothoracic surgery. We present a case of RV perforation presenting shortly after temporary pacing wire removal following primary percutaneous coronary intervention (PPCI) for inferior STEMI at a DGH, managed successfully with bedside diagnosis, stabilization, and prompt regional transfer.

## Summary figure

**Figure:**
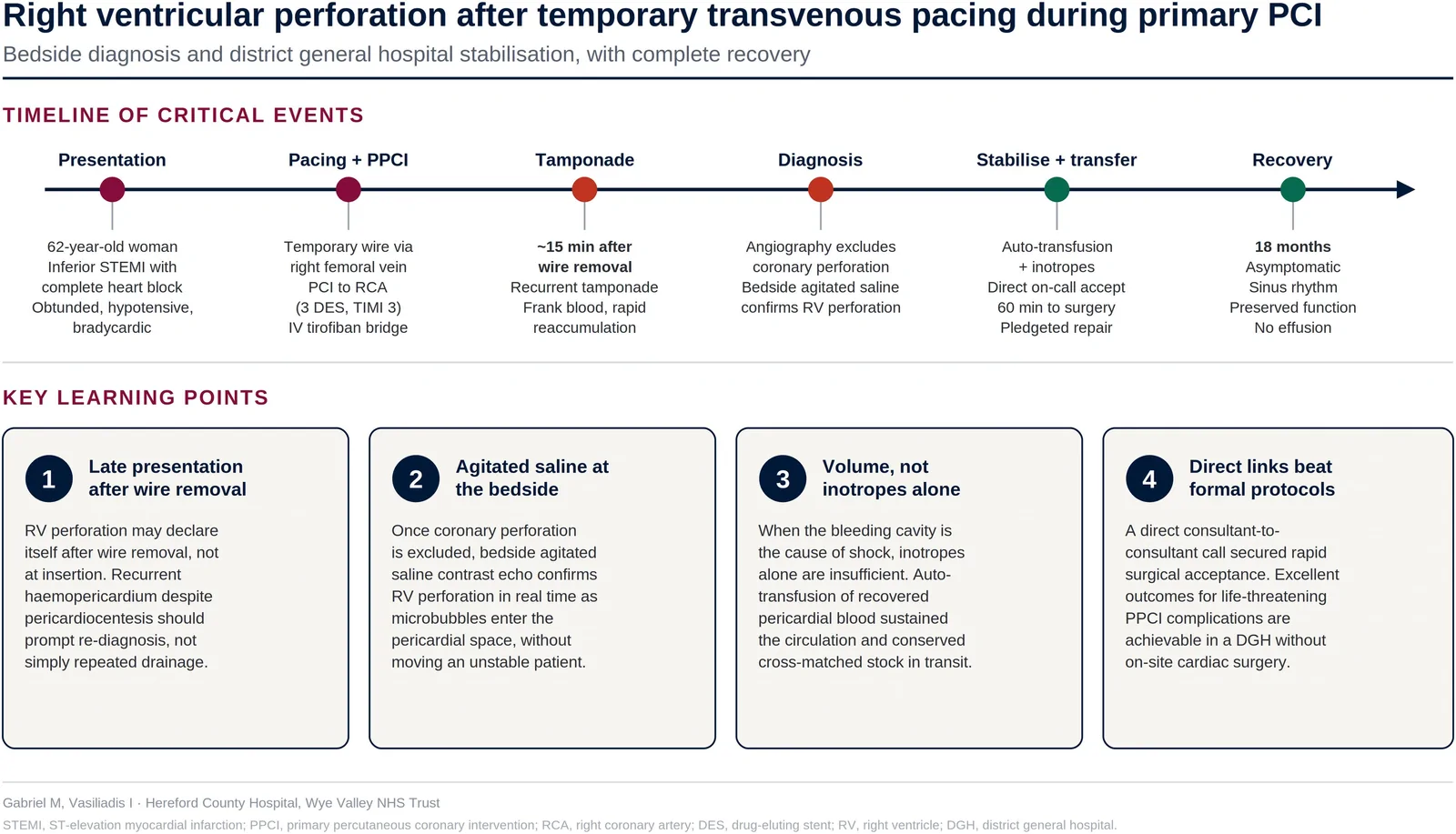
Timeline of critical events from presentation through surgical repair and complete cardiac recovery—see attached file.

## Case presentation

A 62-year-old woman presented with sudden-onset central chest pain and collapse. Her history included hypertension, pre-diabetes, hypercholesterolaemia, obesity (body mass index 33.3 kg/m^2^), and vitamin B12 deficiency, and she smoked 6–8 cigarettes daily. She was antiplatelet, anticoagulant, and statin naïve, taking only amlodipine 10 mg and perindopril 4 mg once daily.

On arrival at the catheterization laboratory, she was obtunded from cerebral hypoperfusion, hypotensive, and bradycardic (37–38 b.p.m.) in complete atrioventricular block with impalpable peripheral pulses. There were no murmurs or gallops, or peripheral oedema, and the chest was clear. Twelve-lead electrocardiography confirmed inferior STEMI with complete atrioventricular block. After failure to respond to maximal intravenous atropine and isoprenaline, an emergency temporary transvenous pacing wire was inserted via the right femoral vein under fluoroscopic guidance, restoring an adequate rate.

Primary percutaneous coronary intervention proceeded immediately after. Periprocedural blood tests showed haemoglobin 86 g/L (reference range: 115–164; attributed to known vitamin B12 deficiency), platelets 274 × 10^9^/L (150–400), creatinine 89 µmol/L (49–90; estimated glomerular filtratation rate 60 mL/min/1.73 m^2^), and potassium 3.8 mmol/L (3.5–5.3). Troponin-I was 36.9 ng/L reflecting the hyperacute phase. Femoral arterial access was chosen given impalpable radial pulses and to preserve large-bore mechanical circulatory support, using the artery ipsilateral to the venous pacing sheath to keep the contralateral groin free. Angiography demonstrated severe diffuse disease of the right coronary artery (RCA) with otherwise minor disease. Primary percutaneous coronary intervention was performed to the RCA with implantation of three overlapping drug-eluting stents from the posterior left ventricular branch to the ostial RCA, achieving TIMI 3 flow with no residual stenosis.

As she was obtunded and unable to swallow safely, oral P2Y12 loading was deferred. She had received aspirin 300 mg pre-hospital and was given weight-adjusted unfractionated heparin, with intravenous tirofiban (glycoprotein IIb/IIIa inhibitor) as a bridging agent. Prasugrel was loaded enterally at the conclusion once her conscious level had recovered and airway protective reflexes were intact. Atrioventricular conduction recovered during revascularization, with pacing spikes disappearing, as the intrinsic rhythm exceeded the backup rate, confirming that the complete atrioventricular block was ischaemic in origin. The pacing wire was removed, and the venous sheath was left *in situ* in case repeat pacing was required.

Approximately 15 min after wire removal, she became hypotensive. Bedside transthoracic echocardiography showed a large circumferential pericardial effusion, ∼1.9 cm in diastole at the apex, with tamponade physiology. Pericardiocentesis with drain insertion yielded frank, non-clotting bright red blood with initial improvement, but the effusion rapidly reaccumulated with recurrent instability. Repeat angiography excluded coronary perforation. Bedside agitated saline contrast echocardiography in subcostal views, with 10 mL of saline injected peripherally, demonstrated echogenic microbubbles within the pericardial effusion, confirming extravasation from the RV cavity (see Supplementary material online, *Video S1*).

Ongoing haemopericardium was managed with repeat aspiration and auto-transfusion. The case was discussed urgently with the on-call tertiary cardiothoracic team who accepted the patient for emergency transfer. Despite no formal transfer protocol, close professional links allowed rapid acceptance by direct consultant-to-consultant discussion. She was stabilized with auto-transfusion and dobutamine throughout a blue-light road transfer, with the pericardial drain on open drainage and aspirated blood re-infused via the venous sheath. Decision-to-arrival time was ∼60 min, including an estimated 40 min of blue-light transfer; cross-matched packed red cells and platelets accompanied the patient, and a urinary catheter was placed prior to departure to monitor end-organ perfusion.

Surgical exploration via median sternotomy at the tertiary centre revealed fresh pericardial blood and a puncture defect in the RV free wall near the apex, consistent with pacing wire-related injury, repaired with two horizontal mattress sutures of 4/0 polypropylene, buttressed with Teflon pledgets to prevent cut-through of the friable infarcted myocardium.

The postoperative course was complicated by transient respiratory failure, transfusion-related acute lung injury, acute kidney injury, and paroxysmal atrial fibrillation, all resolving with supportive care. She was discharged on dual antiplatelet therapy and secondary prevention. At 18 months, she remained asymptomatic in sinus rhythm, with preserved biventricular function (left ventricular ejection fraction > 55%), no residual effusion or ventricular dysfunction, had stopped smoking, and completed cardiac rehabilitation.

## Discussion

Right ventricular perforation is a recognized but uncommon complication of temporary transvenous pacing. In a large US analysis of over 360 000 patients undergoing temporary transvenous pacing, tamponade occurred in 0.6%, with an in-hospital mortality of 14.1%.^2^ Recent prospective data using serial echocardiography in 641 patients identified perforation in 9.2%, with new effusions in 3.7% after electrode insertion, 1.1% before removal, and 2.3% after removal; renal dysfunction and prolonged pacing duration were predictors.^3^ Most perforations are identified at lead insertion and managed conservatively, with only a minority progressing to persistent tamponade requiring surgical repair. When this occurs in a DGH without on-site cardiothoracic services, the management pathway becomes critically important.

This case illustrates several key aspects of successful complication management in the non-tertiary setting. Haemodynamic deterioration occurred after wire removal rather than at insertion, a pattern carrying particular risk of delayed recognition, as initial post-procedural stability can provide false reassurance.^3^ This supports structured post-removal monitoring,^3^ particularly in DGH settings where continuous specialist input may be unavailable. In our case, rapid re-accumulation despite pericardiocentesis prompted systematic diagnostic re-evaluation rather than repeated drainage alone. Repeat angiography excluded coronary perforation; transthoracic echocardiography showed the effusion but not its source, and although CT is highly sensitive for cardiac perforation, it was not feasible in this unstable patient.^4^ Agitated saline microbubbles injected peripherally are normally filtered by the pulmonary circulation and remain confined to the right heart, so their appearance within the pericardial space therefore indicates direct communication from the RV cavity, strongly suggesting perforation. Bedside agitated saline contrast echocardiography, established for shunt detection^5^ and described previously for RV free wall rupture,^6^ thus confirmed the diagnosis. Its principal value here was rapid, point-of-care diagnostic confirmation without moving an unstable patient to imaging that may not be rapidly accessible out-of-hours.

The antithrombotic strategy warrants reflection. The 2023 European Society of Cardiology (ESC) guidelines on acute coronary syndromes (ACS) note that evidence for routine upstream P2Y12 inhibitor pre-treatment is lacking while describing a potential role for glycoprotein IIb/IIIa in high-risk PCI in P2Y12-naïve patients and for intravenous cangrelor (Class IIb) where oral loading is not feasible.^7^ Here, oral P2Y12 therapy was deferred in timing and route rather than withheld: the patient was obtunded and unable to swallow in cardiogenic shock, nasogastric loading risked aspiration, and cangrelor was unavailable locally.^8^ The choice lay between multi-stent PPCI on aspirin monotherapy in an antiplatelet-naïve patient or providing intravenous platelet inhibition as a bridge until enteral P2Y12 loading was safe. The former carried unacceptable stent thrombosis risk on a complex three-stent construct. Intravenous tirofiban was therefore used as a bridge, with prasugrel loaded enterally once conscious level and airway protective reflexes had recovered. Nevertheless, we acknowledge that this potent platelet inhibition may have amplified the haemopericardium, an association reported previously in patients receiving glycoprotein IIb/IIIa inhibitors during temporary transvenous pacing, where three of four developed tamponade after wire removal or manipulation.^9^

Finally, this case highlights the importance of systems-level preparedness in DGHs delivering PPCI without co-located cardiothoracic surgery, which the 2023 ESC ACS guidelines recommend be supported by established transfer protocols.^7^ A favourable outcome was nonetheless achieved, despite the absence of a formal protocol because several elements aligned: rapid recognition and re-evaluation on the table, active stabilization for transfer with continuous inotropes, an open pericardial drain, and intermittent manual auto-transfusion, all accompanied by cross-matched products and a critical care escort. Re-infusion of recovered autologous pericardial blood is a simple means of sustaining the circulation when surgery is not immediately available. The decisive factors were not a written protocol, but the capacity to recognize the complication, confirm it at the bedside, stabilize aggressively, and escalate rapidly. We would argue that every PPCI-capable DGH should ensure its on-call teams can replicate these steps, and standing transfer agreements would further reduce time to surgery. Limitations include that this is a single-case report, the echocardiographic images were captured from bedside recordings during an acute emergency, and generalizability depends on the availability of regional transfer networks.

## Conclusion

Right ventricular perforation associated with temporary pacing may present with tamponade shortly after wire removal rather than at insertion. Once coronary perforation is excluded, bedside agitated saline contrast echocardiography offers rapid, low-cost, point-of-care confirmation. Excellent outcomes remain achievable in DGHs without on-site cardiothoracic surgery when systematic re-evaluation, multi-faceted haemodynamic stabilization including auto-transfusion and inotropic support, judicious antithrombotic decision-making, and close professional links with a regional tertiary cardiothoracic centre are in place.

## Supplementary Material

ytag590_Supplementary_Data

## Data Availability

No new data were generated or analysed in support of this case report.

## Lead author biography

Maria Gabriel graduated from Norwich Medical School, University of East Anglia, in 2024 with an MBBS degree. She is currently a Foundation Year 2 doctor in the Department of Cardiology at Hereford County Hospital, Wye Valley NHS Trust. She has a strong interest in cardiology and acute medicine, with experience in quality improvement, teaching, and research, including national and international presentations. She is preparing to pursue a career in cardiology through internal medicine training.

## Notes

**Consent:** The authors confirm that written informed consent was obtained from the patient for publication of this case report, including any associated images, videos, and data, in accordance with the Committee on Publication Ethics (COPE) guidelines.

## References

[1] Glikson M, Nielsen JC, Kronborg MB, Michowitz Y, Auricchio A, Barbash IM, et al 2021 ESC guidelines on cardiac pacing and cardiac resynchronization therapy. Eur Heart J 2021;42:3427–3520. 34455430 10.1093/eurheartj/ehab364

[2] Metkus TS, Schulman SP, Marine JE, Eid SM. Complications and outcomes of temporary transvenous pacing: an analysis of >360,000 patients from the national inpatient sample. Chest 2019;155:749–757. 30543806 10.1016/j.chest.2018.11.026

[3] Dwivedi SK, Sharma A, Chaudhary G, Chandra S, Bhandari M, Vishwakarma P, et al Incidence and predictors of temporary pacemaker implantation–induced cardiac perforation. Heart Rhythm 2025;22:e1000–e1006. 40582687 10.1016/j.hrthm.2025.06.044

[4] Waddingham PH, Elliott J, Bates A, Bilham J, Muthumala A, Honarbakhsh S, et al Iatrogenic cardiac perforation due to pacemaker and defibrillator leads: a contemporary multicentre experience. EP Europace 2022;24:1824–1833. 10.1093/europace/euac105 35894862

[5] Bernard S, Churchill TW, Namasivayam M, Bertrand PB. Agitated saline contrast echocardiography in the identification of intra- and extracardiac shunts: connecting the dots. J Am Soc Echocardiogr 2021;34:1–12. 10.1016/j.echo.2020.09.013 34756394

[6] Ibrahem A, Farag M, Elzein H, Dundas J, Ramesh B, Egred M. Right ventricular free wall rupture complicating anterior STEMI. JACC Case Rep 2022;4:770–774. 35818593 10.1016/j.jaccas.2022.04.028 PMC9270625

[7] Byrne RA, Rossello X, Coughlan JJ, Barbato E, Berry C, Chieffo A, et al 2023 ESC guidelines for the management of acute coronary syndromes. Eur Heart J 2023;44:3720–3826. 37622654 10.1093/eurheartj/ehad191

[8] Ferlini M, Raone L, Bendotti S, Currao A, Primi R, Bongiorno A, et al Cangrelor in patients undergoing percutaneous coronary intervention after out-of-hospital cardiac arrest. J Clin Med 2024;14:76. 39797159 10.3390/jcm14010076 PMC11722389

[9] Jeilan M, Richardson G, Gershlick A. Transvenous pacing causing tamponade in patients receiving glycoprotein IIb/IIIa inhibitors for percutaneous coronary intervention. J Invasive Cardiol 2007;19:E40–E42. 17268050

