# Supplementary material for: Successful management of cardiac tamponade due to right ventricular perforation following temporary pacing wire removal after primary percutaneous coronary intervention at a district general hospital: a case report
Source: Eur Heart J Case Rep. 2026 Aug 8;10(8):ytag590. doi: 10.1093/ehjcr/ytag590 (PMC13496324; doi:10.1093/ehjcr/ytag590)
Supplement: Supplementary file 1 [file ytag590_supplementary_data.zip › Supplemental video legend.docx]

**Video 1.** Bedside transthoracic echocardiography with agitated saline contrast study performed in subcostal views in the cardiac catheterisation laboratory during the acute presentation. The video demonstrates a large circumferential pericardial effusion (maximum approximately 1.9 cm in diastole at the apex). Following agitated saline contrast injection via the venous sheath, echogenic microbubbles become visible within the pericardial effusion, confirming real-time extravasation from the right ventricular cavity into the pericardial space through the perforation site, consistent with right ventricular perforation related to the temporary pacing wire.

*Alt text: a bedside echocardiography video recorded from the ultrasound screen in subcostal view. The video shows cardiac chambers surrounded by a large dark pericardial effusion. After agitated saline contrast injection, bright echogenic microbubbles appear progressively within the pericardial fluid space outside the heart, demonstrating extravasation from the right ventricular cavity through the perforation site into the pericardium.*
